# Supplementary material for: Transparent ultrahigh-molecular-weight polyethylene/MXene films with efficient UV-absorption for thermal management
Source: Nat Commun. 2024 Apr 9;15:3076. doi: 10.1038/s41467-024-47432-z (PMC11004134; doi:10.1038/s41467-024-47432-z)
Supplement: Supplementary file 1 — Supplementary Information [file 41467_2024_47432_MOESM1_ESM.pdf]

Supporting Information

Transparent ultrahigh-molecular-weight polyethylene/MXene films with efficient UV absorption for thermal management

Xianhu Liu[1,2#], Wenrui Zhang[1,2#], Xin Zhang[1], Zhengui Zhou[3], Chunfeng Wang[2,4], Yamin Pan[1,*], Bin Hu[3,*], Chuntai Liu[1, *], Caofeng Pan[2,5*], Changyu Shen[1]

[1]College of Materials Science and Engineering, State Key Laboratory of Structural Analysis, Optimization and CAE Software for Industrial Equipment, National Engineering Research Center for Advanced Polymer Processing Technology, Zhengzhou University, Zhengzhou, 450002, China

[2] Beijing Institute of Nanoenergy and Nanosystems, Chinese Academy of Sciences, Beijing 101400, P. R. China

[3] Wuhan National Laboratory for Optoelectronics, Huazhong University of Science and Technology, Wuhan 430074, P. R. China

[4] College of Physics and Optoelectronic Engineering, Shenzhen University, Shenzhen 518060, P. R. China

[5] Institute of Atomic Manufacturing, Beihang University, Beijing 100191, China

[#]These authors contributed equally: Xianhu Liu, and Wenrui Zhang.

[*]Corresponding authors.

E-mail:yamin.pan@zzu.edu.cn;bin.hu@hust.edu.cn;ctliu@zzu.edu.cn; cfpan@binn.cas.cn

Supplementary Note 1. Detailed steps for the measurement of Haze

Haze is the percentage of the intensity of transmitted light deviated from the incident light at an angle of 2.5° or above in the total transmitted light intensity. The greater the haze means that the film luster and transparency decrease. Our haze measurement was done according to ASTM D1003 "Standard Method for Haze and Luminous Transmittance of Transparent Plastics", which is defined as:

$$Haze = (\frac{T_4}{T_2} - \frac{T_3}{T_1}) \times 100\%$$

where T_4 is scattered luminous flux of instruments and films; T_3 is scattered luminous flux of instruments; T_2 is luminous flux through films; T_1 is incident luminous flux. The haze value is calculated from the value of T_1, T_2, T_3 and T_4 measured by the UV-Vis photometer.

According to this principle, four-step method was used to measure the film haze. The first step is to correct the background by placing a white plate in the reflectance port; the second step is to place the film at the transmission port while keeping the position of the white plate unchanged to measure the total luminous transmittance; the third step is to remove the white plate and measure the light scattering rate of the sample and instrument; finally, the film and the white plate are removed to obtain the scattering of the instrument. The haze of the film can be obtained by calculation of the value obtained from the above four steps. The whole testing process and data processing are carried out by the machine to eliminate external interference. A concise step-by-step description of the measurement is summarized in Supplementary Table 2.

Supplementary Note 2. Detailed description for accelerated aging test

The composite films were exposed to UV radiation (UVA-340) in an accelerated aging chamber (Linpin). Test conditions of UVA radiation at 60 °C of 0.72 W/m^2 for a period of 3 days (72 h) were employed. Test was conducted according to ASTMF 1980:2002 "Standard Guide for Accelerated Aging of Sterile Medical Device Packages".

According to ASTMF 1980:2002, at the UVA-340 irradiance setting value of 0.72 W/m^2, the total radiation intensity of the UV lamp is 1186 W/m^2. In Beijing, China, for example, its annual average hourly solar radiation[1] is about 171 W/m^2. Therefore, the ultraviolet acceleration factor (UV_{AF}) of the accelerated aging test is 6.94. The acceleration factor of temperature (T_{AF}) is calculated by the Arrhenius model as follows:

$$T_{AF} = exp\left[\frac{E_a}{k} \times \left(\frac{1}{T_{normal}} - \frac{1}{T_{stress}}\right)\right]$$

where T_{normal} is room temperature, T_{stress} is testing temperature, E_a is failure reaction activation energy (eV), and k is Boltzmann constant (eV/K). Thus, T_{AF} of the accelerated aging test is 10.43.

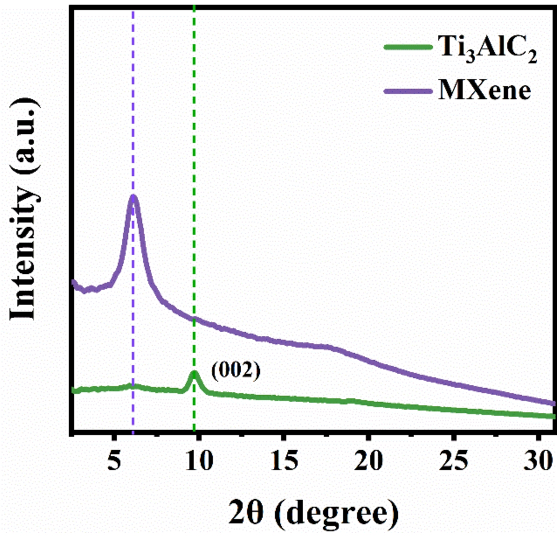

Supplementary Fig. 1 1D-WAXD spectra of Ti_3AlC_2 and MXene.

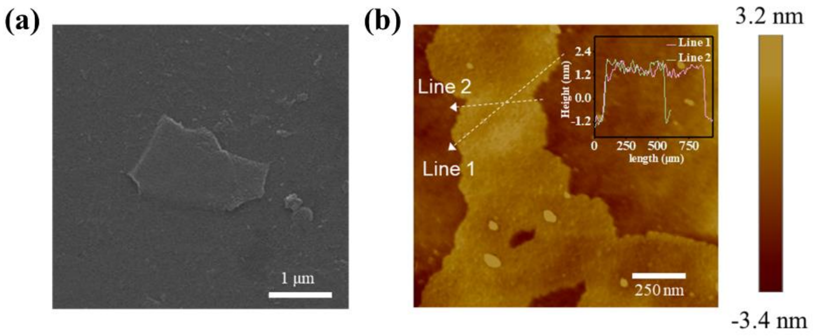

Supplementary Fig. 2 Structural characterization of the MXene. (a) SEM image of a $Ti_3C_2T_x$ MXene nanosheet. **(b)** AFM image of stripped $Ti_3C_2T_x$ MXene nanosheets with an illustration showing the height profile along the indicated dashed lines.

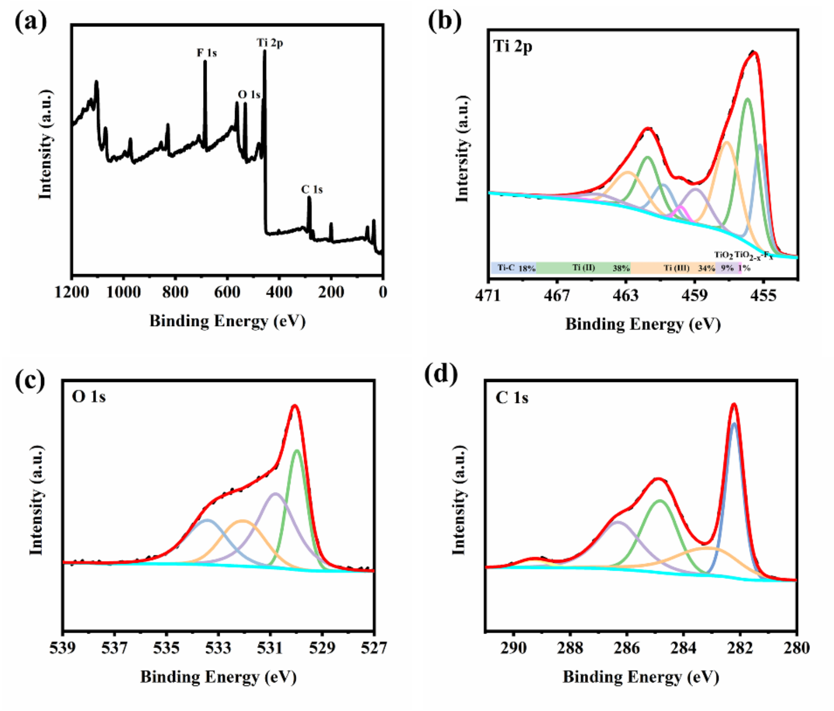

Supplementary Fig. 3 (a) XPS survey spectra of MXene. High-resolution XPS spectra of the **(b)** Ti 2p, **(c)** O 1s, and **(d)** C 1s peaks for MXene.

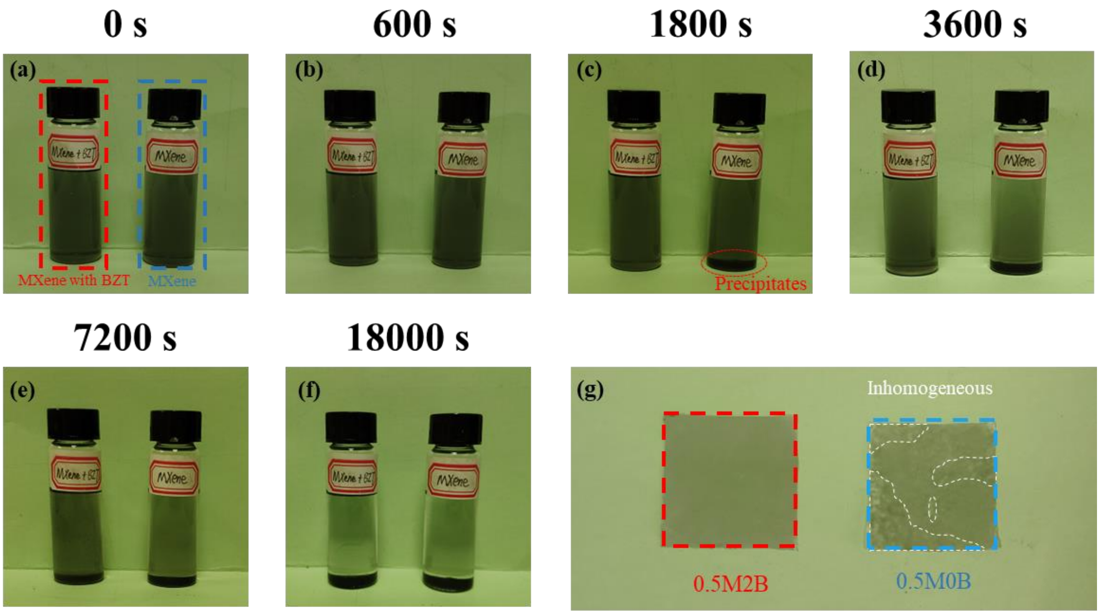

Supplementary Fig. 4 Dispersion of MXene with or without BZT. (a)-(f) Photographs of dispersion of MXene alone (right) in xylene and after addition of BZT (left). **(g)** Photograph of the composite film before compression of 0.5M2B and 0.5M0B.

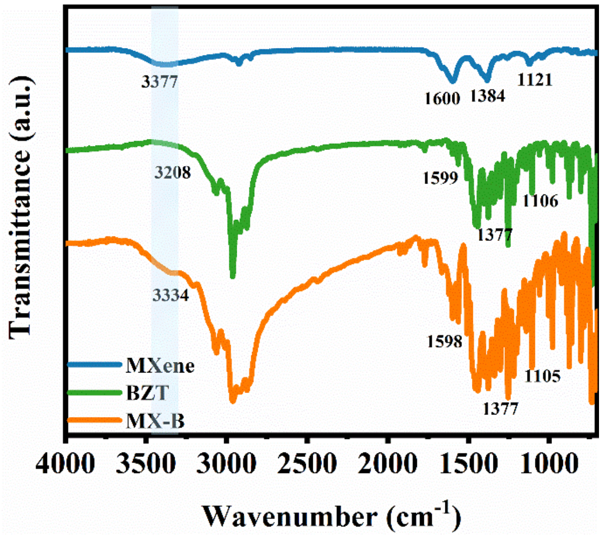

Supplementary Fig. 5 FTIR spectra of MXene, BZT powder and both together.

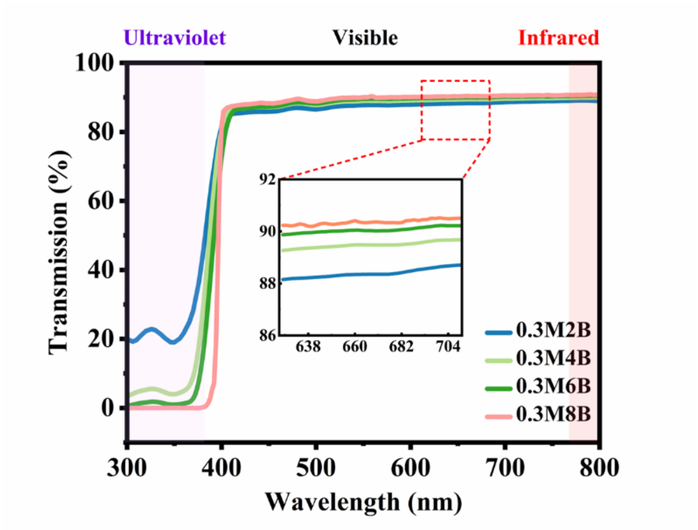

Supplementary Fig. 6 Optical properties of films with 0.3 wt.% MXene and different contents of BZT.

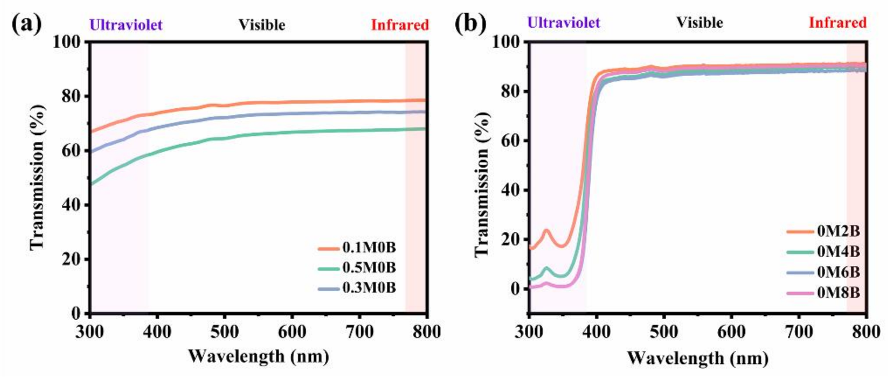

Supplementary Fig. 7 Optical properties of films containing MXene **(a)** or BZT **(b)** alone.

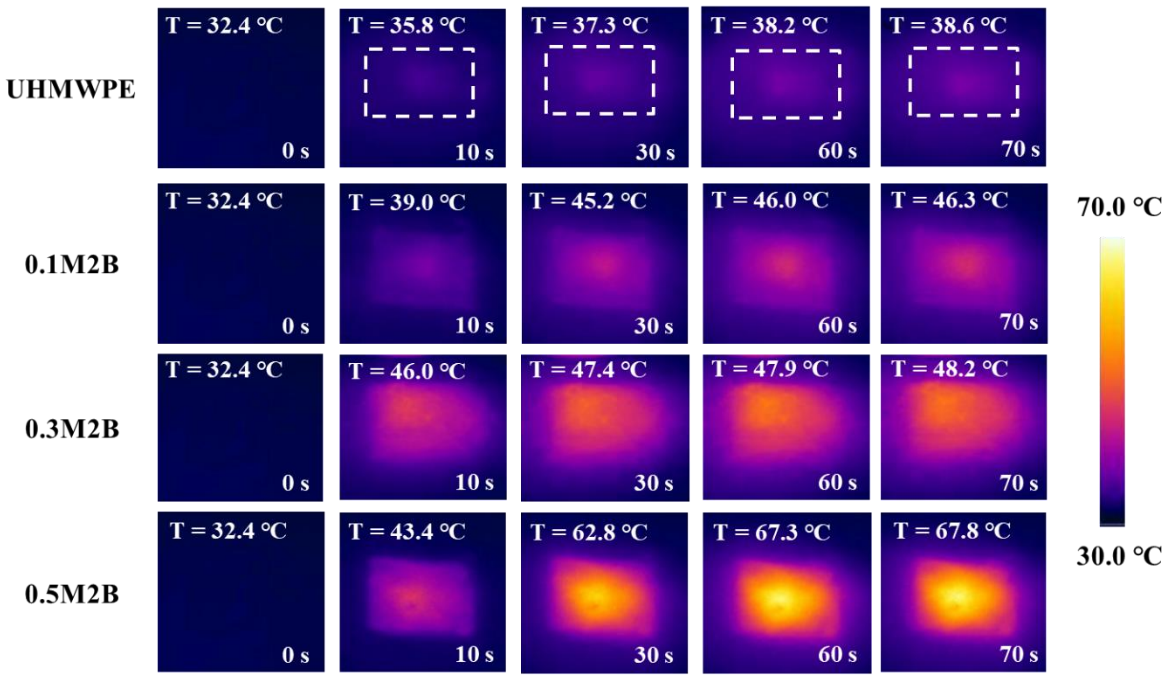

Supplementary Fig. 8 IR images of UHMWPE films with 2 wt.% BZT and different MXene contents irradiated under 400 mW cm^{-2}.

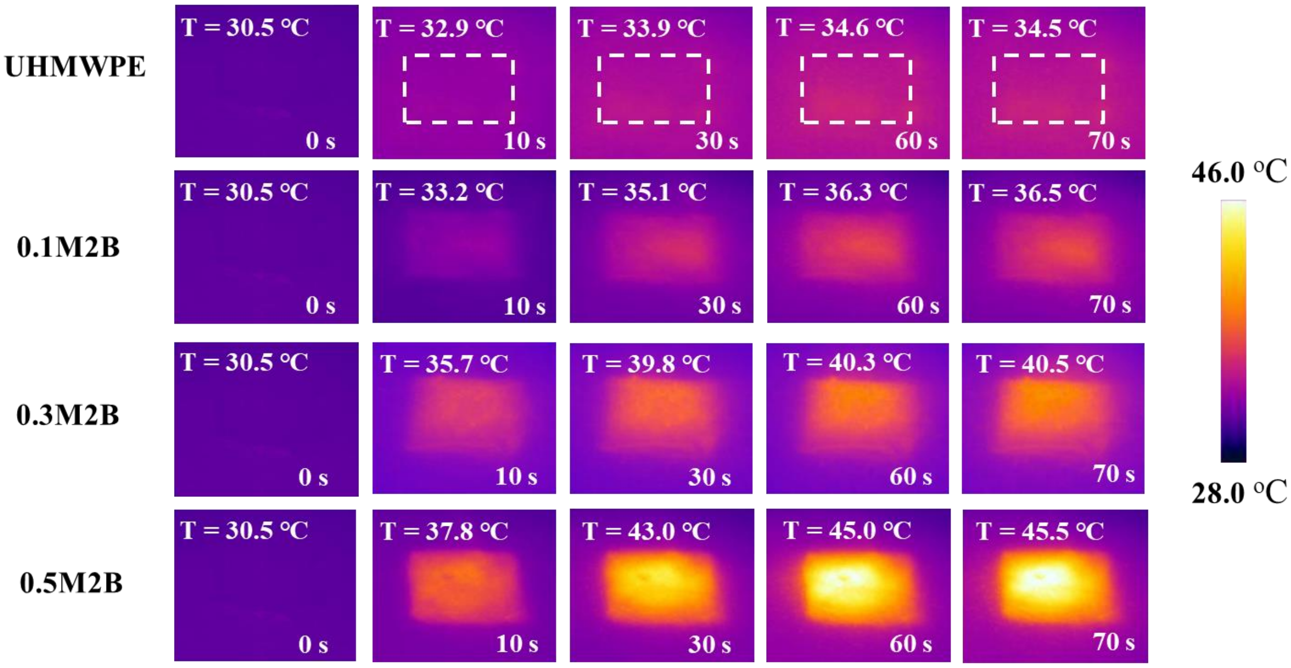

Supplementary Fig. 9 IR images of UHMWPE films with 2 wt.% BZT and different MXene contents irradiated under 100 mW cm^{-2}.

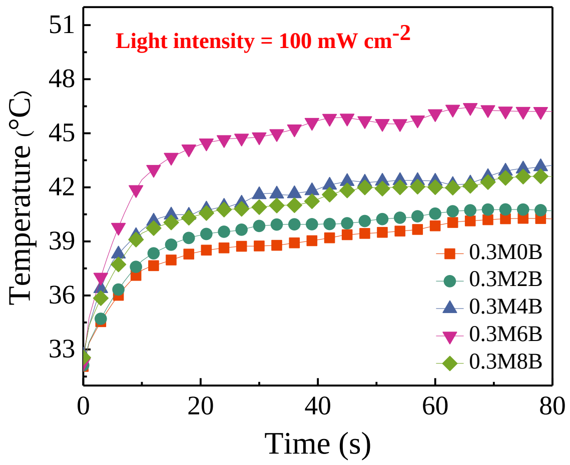

Supplementary Fig. 10 Temperature-time curve of composites films with 0.3 wt.% MXene and different content of BZT under light irradiation of 100 mW cm^{-2}.

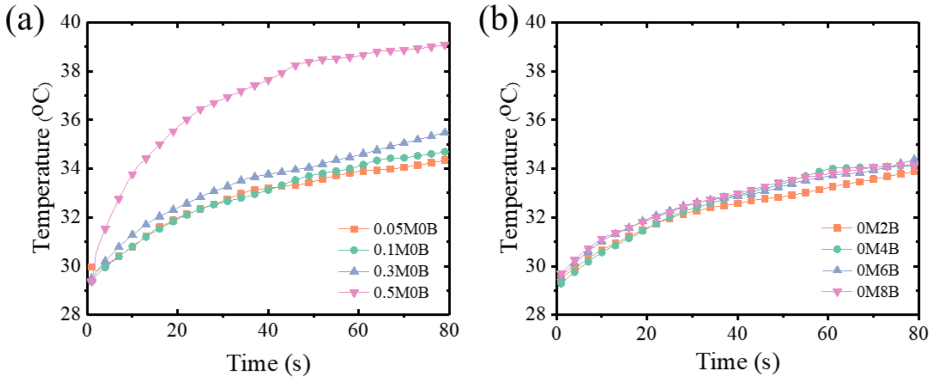

Supplementary Fig. 11 Photothermal conversion of film in control group. Temperature-time curve of composites films with different content of MXene **(a)** and different content of BZT **(b)** under 100 mW cm^{-2}.

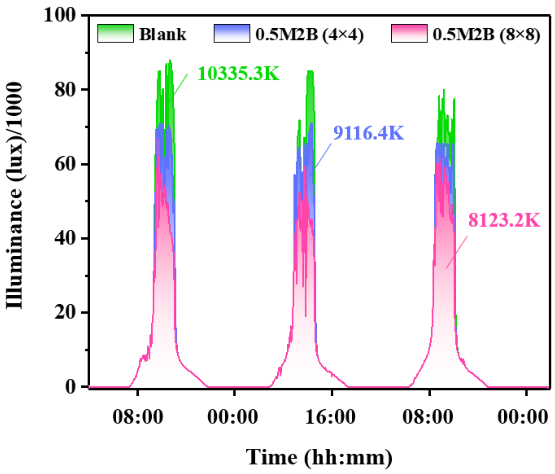

Supplementary Fig. 12 Variation of illumination over time inside a cube container in Zhengzhou, China.

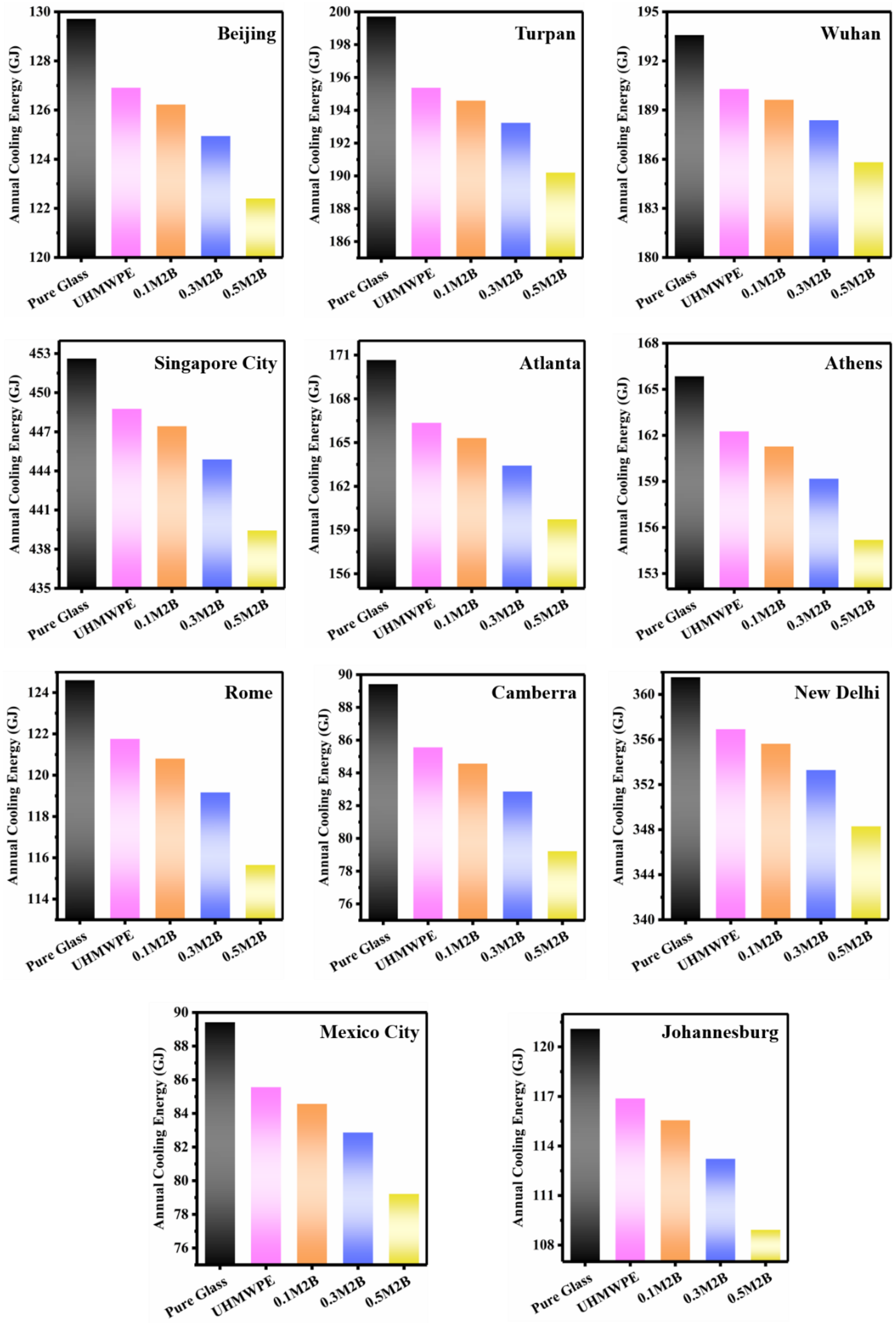

Supplementary Fig. 13 The annual cooling energy consumption of building models using these four types of windows based on weather data from 11 cities.

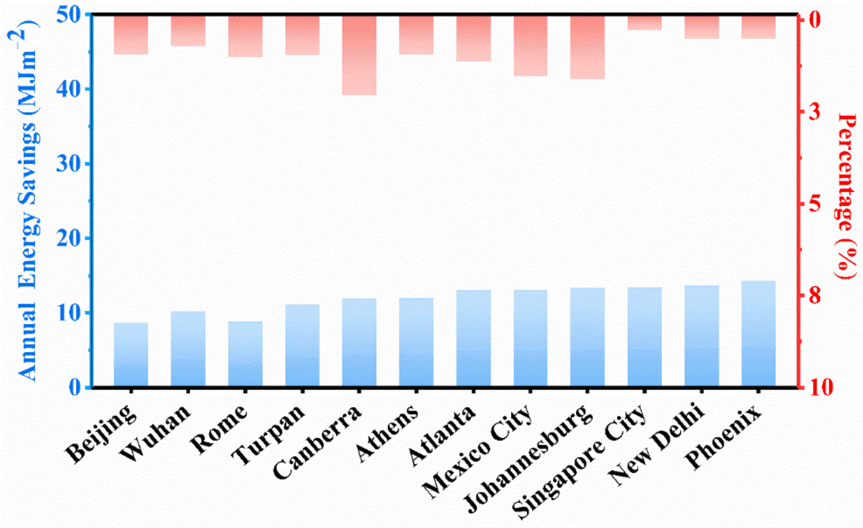

Supplementary Fig. 14 The annual cooling energy savings and percentage of building models using UHMWPE film based on weather data from 12 cities.

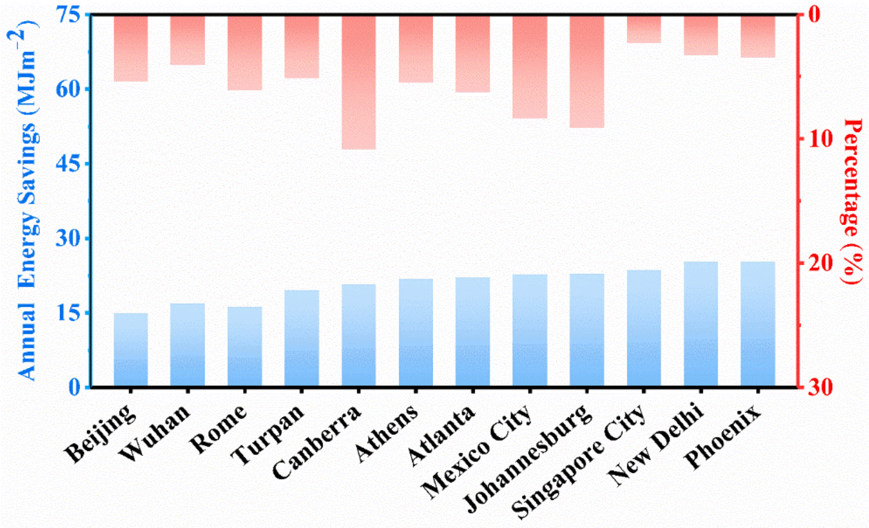

Supplementary Fig. 15 The annual cooling energy savings and percentage of building models using 0.1M2B film based on weather data from 12 cities.

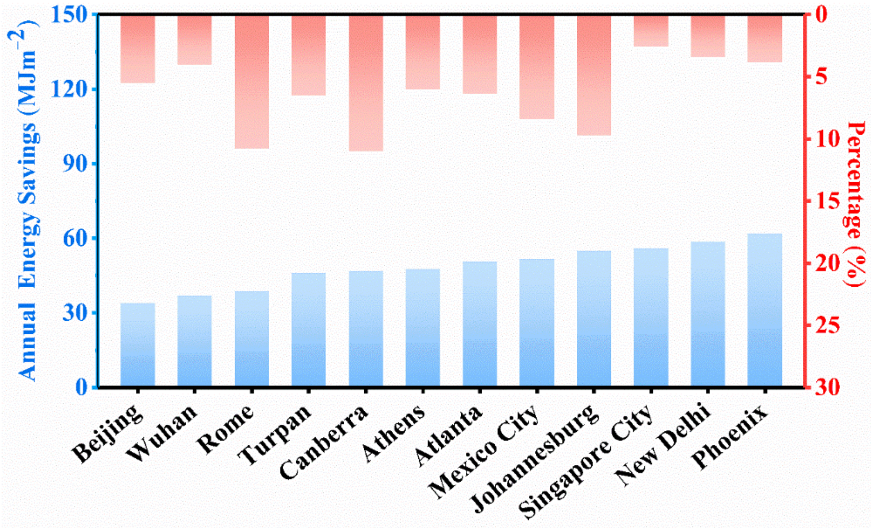

Supplementary Fig. 16 The annual cooling energy savings and percentage of building models using 0.3M2B film based on weather data from 12 cities.

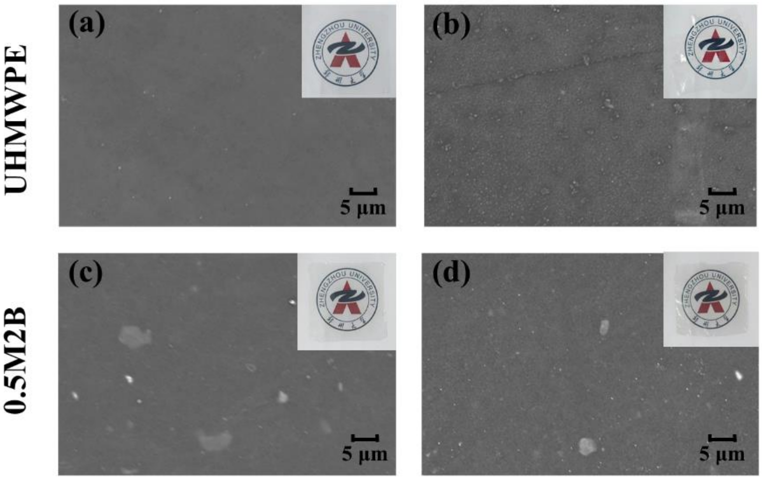

Supplementary Fig. 17 Optical photographs and SEM images of composite films before (left) and after (right) 72 hours accelerated aging test.

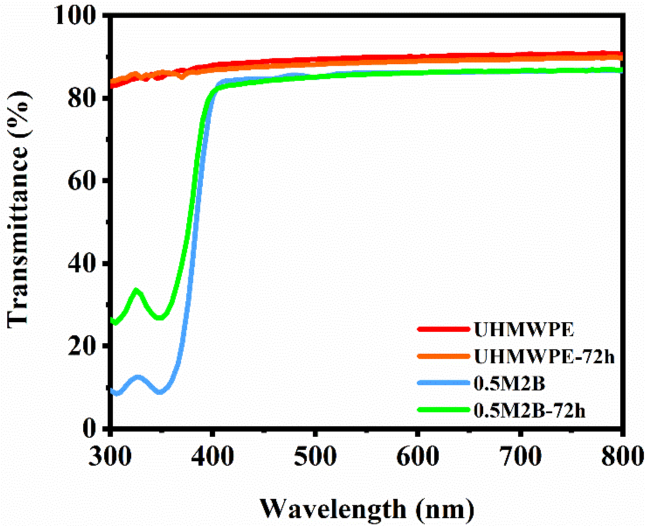

Supplementary Fig. 18 UV-vis transmission spectra of composite films before and after 72 hours accelerated aging test.

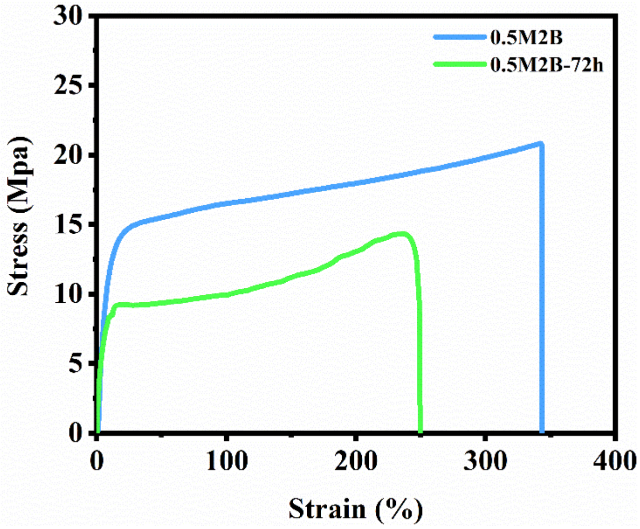

Supplementary Fig. 19 Stress-strain curves for 0.5M2B film before and after 72 hours accelerated aging test.

Supplementary Table 1 The thickness of UHMWPE and MXene@BZT/UHMWPE films before and after pressing.

Sample		UHMWPE	0.05M2B	0.1M2B	0.3M2B	0.5M2B	0.3M0B	0.3M4B	0.3M6B	0.3M8B
Film Thickness (μm)	Before	38	35	41	35	33	35	36	36	38
	After	20	21	24	22	18	21	22	19	22

Supplementary Table 2 Four-step method for measuring haze

Step	Description	Purpose
1	No sample, white plate in reflectance port	Background calibration
2	Sample in transmittance position, white plate in reflectance port	Total luminous transmittance
3	Sample in position, light trap in reflectance port	Sample and instrument scatter
4	No sample, no white plate	Instrument scatter

Supplementary Table 3 The transmittance and haze of the composite film are compared with other polyethylene-based composite materials.

Composites	Transmission (%)	Haze (%)	Thickness (μm)	Years	Ref
Green LDPE	52.0 ± 5.2	67.9 ± 0.5	150	2020	2
Green LDPE/0.1 wt.% GC	48.9 ± 1.9	78.8 ± 0.4			
Green LDPE/0.3 wt.% GC	46.5 ± 4.0	89.2 ± 1.6			
Green LDPE/0.5 wt% GC	41.5 ± 1.7	95.4 ± 0.6			
20% LLDPE/80% HDPE	~91	~26.8	100	2020	3
40% LLDPE/60% HDPE	~91.5	~25			
10% PP/30% LLDPE/60% HDPE	~91.5	~23.7			

UHMWPE-undraw	~70	~91	/	2019	4
UHMWPE-20x	~80	~64	/	2019	4
UHMWPE-60x	~81	~50			
PE/5 wt.% CNF	~80	~53	130	2017	5
PE/10 wt.% CNF	~75	~68			
PE/20 wt.% CNF	~70	~80			
PE/30 wt.% CNF	~53	~95			
PE/5 wt.% EMAA	~88	~40	112	2019	6
PE/10 wt.% EMAA	~89	~37.5	82		
PE/20 wt.% EMAA	~89.5	~53	44		

PE/30 wt.% EMAA	~89.8	~67.5	28	
**This work**	**85~92**	**12~30**	**19~24**	**This work**

Green LDPE/GC: green low-density polyethylene/glassy carbon; LLDPE/HDPE: linear low-density polyethylene/high-density polyethylene; PE/CNF: polyethylene/cellulose nanofibril; EMAA: ethylene/methacrylic acid

Supplementary Table 4 Geographical coordinates and climate types of 12 cities

City	Geographical coordinates	Climatic zones	Climatic types
Beijing (China)	39º48′N, 116º28′E		Monsoon climate of medium latitudes
Turpan (China)	42º55′N, 89º12′E	Temperate zone	Temperate continental climate
Canberra (Australian)	35º18′S, 149º10′E		Temperate oceanic climate
Wuhan (China)	30º37′N, 114º7′E		Subtropical monsoon climate
Rome (Italy)	41 º 48′N, 12º13′E		Mediterranean climate
Athens (Greece)	34º54′N, 27º43′E	Subtropical zone	Mediterranean climate
Atlanta (USA)	33º37′N, 84º25′W		Subtropical monsoon climate
Mexico City (Mexico)	19º25′N, 99º4′W		Subtropical plateau climate

Johannesburg (South Africa)	26º7′S, 28º13′E	Subtropical zone	Subtropical plateau climate
Singapore City (Singapore)	1º1′N, 103º3′E	Tropical zone	Tropical rainy climate
New Delhi (India)	28º34′N, 77º12′E		Tropical monsoon climate
Phoenix (USA)	33º27′N, 111º58′W		Tropical desert climate

REFERENCES

1 China Meteorological Data Service Center. http://data.cma.cn/Market/Detail/code/RADI_CHN_MUL_HOR/type/0.html

2 Oyama, I. C. *et al*. A new eco-friendly green composite for antistatic packaging: Green low-density polyethylene/glassy carbon. *Polym. Compos.* **41**, 2744-2752 (2020).

3 Wu, W., Wang, Y. Modified high-density polyethylene films: preparation, composition and their physical properties. *Bull. Mat. Sci.* **43**, 143 (2020).

4 Lozano L. M. *et al*. Optical engineering of polymer materials and composites for simultaneous color and thermal management. *Opt. Mater. Express* **9**, 1990-2005 (2019).

5 Maia T. H. S. *et al*. Polyethylene cellulose nanofibrils nanocomposites *Carbohydr. Polym.* **173**, 50-56 (2017).

6 Ali S. *et al*. Preparation of polyethylene and ethylene/methacrylic acid copolymer blend films with tunable surface properties through manipulating processing parameters during film blowing. *Polymers* **11**, 1565 (2019).
